# The RNA-Binding Protein ELAVL4/HuD Promotes Stress-Associated Tumor Survival in Neuroendocrine Cancers

**DOI:** 10.3390/ijms27177882

**Published:** 2026-09-03

**Authors:** Kausik Bishayee, Vaishak Kaviyarasan, Sun Young Yoo, Seung Hee Lee, Yong Soo Park

**Affiliations:** Department of Anatomy, College of Medicine, The Catholic University of Korea, Seoul 06591, Republic of Korea; vikash.vaishak777@gmail.com (V.K.); easy11@catholic.ac.kr (S.Y.Y.); yongsoopark88@catholic.ac.kr (Y.S.P.)

**Keywords:** ELAVL4/HuD, GRB-10, ARL6IP1, mTORC1, cancer cell survival

## Abstract

Neuro-endocrine-related cancers overexpress ELAVL4 (HuD), a neuron-specific RBP implicated in post-transcriptional regulation. HuD plays a role in tumor cell survival; its knockdown in cancer cells and tumors leads to reduced growth and viability. In this review, we addressed HuD’s role in several molecular pathways that contribute to the development of cancer. For example, cancer cells that have high levels of HuD are able to better withstand stressful tumor microenvironments because HuD stabilizes certain mRNAs, which promotes tumor development, modulates oxidative and metabolic stress, enhances autophagy, and impairs apoptosis. We discussed future studies on HuD in cancer, which should focus on its diverse roles in various cancer types and the molecular aspects. Furthermore, assessing HuD’s contribution to the efficacy of immunotherapy requires an understanding of how it affects immune responses in malignancies. The development of targeted therapies, such as RNA-based approaches and small molecules, offers intriguing avenues for precision cancer management. The transition of HuD-directed therapies from preclinical models to clinical trials is anticipated to be accelerated by ongoing advancements in RNA-targeted drug discovery, structural biology, medicinal chemistry, and targeted drug delivery, even though no HuD-specific therapies have yet entered standard clinical practice.

## 1. Introduction

Mammalian gene expression involves a complex and tightly controlled process that includes transcription, mRNA processing, transport, stability, and translation [1]. This regulation is influenced by RNA-binding proteins (RBPs) and unconventional RBPs. RBPs interact with specific mRNA sequences and can bind to single- or double-stranded RNA, playing a key role in various RNA functions that ultimately affect cell fate [2]. They protect target RNAs in the nucleus, ensuring effective nuclear export and completion of translation in the cytoplasm [3]. Over thousands of unique RBPs in humans and mice play functions in processes such as mRNA maturation, stabilization of protein complexes, alternative splicing, polyadenylation, and mRNA degradation [4]. Dysfunction of RBPs is linked to several diseases, including cardiovascular issues, immune disorders, cancer, and neurodegenerative diseases, particularly affecting conditions of the central nervous system like Alzheimer’s and Parkinson’s diseases [5].

The ELAV (embryonic lethal abnormal vision)/Hu family includes unique RBPs required for neurogenesis and post-transcriptional regulation. They stabilize mRNA and regulate splicing and translation, particularly in brain tissues. Mammalian members (ELAVL1 (HuR), ELAVL2 (HuB), ELAVL3 (HuC), and ELAVL4 (HuD)) play an important role in neural growth and development as well as in neurological diseases and various cancers [5]. These proteins influence stability, transport, and translation of mRNAs by binding to the AU-rich elements (AREs) in the 3′ untranslated regions (UTRs). Binding with the 3′-UTR of the target mRNAs to influence the expression of important molecules like cytokines and growth factors [6]. This interaction can help stabilize mRNA and prevent its degradation. Hu proteins are essential for co-transcriptional regulation of pre-mRNA and vital for neuronal development. Their mutations can lead to severe defects. Hu proteins in humans have also been identified as tumor-specific antigens linked to neurological disorders resulting from anti-Hu antibodies that penetrate the blood–brain barrier [5,7,8]. In mammals, Hu proteins can be found in different areas of the cell, such as the nucleus or cytoplasm, depending on factors like cell type and stress [9]. For instance, HuR is mainly found in the nucleus but can shift to the cytoplasm when the cell is under stress. In contrast, HuD is mostly located in the cytoplasm, particularly in neuronal axons and synapses [5]. These proteins play important roles in regulating mRNA due to their flexible localization. Hu- proteins feature three conserved RNA recognition motifs (RRMs). The first two RRMs are closely associated at one end, while the third RRM is connected to a site that recognizes polyadenylation, allowing these proteins to shuttle between the nucleus and cytoplasm [10,11].

The Hu family can form multimers with each other and interact with various proteins [12,13]. HuD promotes mRNA translation by binding to the Eukaryotic Translation Initiation Factor 4A1 (eIF4A) [14]. HuD interacts with the light chain of the microtubule-associated protein MAP1B [15] and with TAP/NXF1 [16], which helps export bulk mRNA. It also interacts with small non-coding RNA Y3 to regulate its RNA targets [17]. In neurons, HuD levels are influenced by micro-RNA miR375, which is controlled by Neurogenic Differentiation Factor 1 (NeuroD1) [18,19]. Research has primarily focused on RNA substrates for HuD in neurons and non-transformed cells. The functions of these proteins are increasingly associated with various diseases, suggesting a need for more research into their roles in neurological development and disorders, cancer, and other health issues. In this review, we will discuss one important neuron-related Hu-family member, HuD, and its activity in certain cancers and in normal physiological conditions.

HuD, in neuroendocrine tumors, maintains tumor resilience in unfavorable microenvironmental circumstances. In this review, we integrate the structural features, post-transcriptional regulatory cascades, and stress-adaptive pathways—particularly autophagy, apoptotic blockade, and metabolic reprogramming—while convincingly linking these molecular events to clinically relevant outcomes such as therapy resistance, metastatic behavior, and immune-related paraneoplastic manifestations. Furthermore, we discussed the contextual duality of HuD function and provided a forward-looking evaluation of emerging therapeutic avenues, such as RNA-based interference and targeted protein degradation, thus providing a valuable conceptual framework that will inform both mechanistic exploration and translational strategies in this difficult cancer subset.

## 2. HuD in Neuroendocrine Neoplasms

HuD is intricately connected to neuroendocrine tumors (NETs) and highly aggressive neuroendocrine carcinomas (NECs) due to the shared biological features between neuroendocrine tissues and the nervous system. Neuroendocrine neoplasms are broadly classified by the World Health Organization (WHO) into well-differentiated neuroendocrine tumors (NETs, graded G1 to G3 based on mitotic rates and Ki-67 proliferation indexes) and poorly differentiated neuroendocrine carcinomas (NECs, always high-grade and defined by TP53 and RB1 mutations) [20]. This histological divide directly dictates clinical prognosis; data from comprehensive population studies indicate that patients with low-to-intermediate grade NETs often experience an indolent disease course with long-term survival rates exceeding 80%, whereas those diagnosed with high-grade NECs face an aggressive, rapidly metastatic trajectory where survival times drop precipitously [21]. Overcoming treatment resistance remains a major hurdle in clinical oncology because tumors routinely escape somatostatin receptor (SSTR)-targeted therapies and peptide receptor radionuclide therapy (PRRT) through receptor downregulation and cellular heterogeneity. Furthermore, long-term exposure to small-molecule inhibitors triggers resistance via alternative pathway crosstalk (such as PI3K/AKT/mTOR loops), while systemic clonal evolution can cause slow-growing NETs initially to accumulate genomic alterations and transform into aggressive, therapy-refractory high-grade phenotypes [22].

## 3. Molecular Structure and Generalized Functions of HuD

RNA-binding protein regulates how mRNAs are processed once they are produced. In general, HuD modulates alternative splicing, mRNA stability, and protein translation [17,23]. This protein binds to specific AU-rich areas in the 3′ untranslated region of several target mRNAs, including GAP43, VEGF, FOS, CDKN1A, and ACHE [24,25]. Many of these target mRNAs encode proteins responsible for RNA binding, transcription control, and neuronal development. HuD lowers mRNA de-adenylation by attaching to the 3′ UTR, thereby stabilizing and preventing their disintegration [26]. It also attaches to the mRNA’s polyadenylated tail, which improves its capacity to interact with other mRNAs [26,27]. This RNA-binding protein predominantly affects neuron-focused RNA processing by stabilizing key mRNAs.

In humans, the gene HuD is located on chromosome 1p34, while in mice, it is on chromosome 4 [5]. It has seven coding exons (E2 to E8) and plays an important role in brain development and function [7,28]. HuD’s mRNA is detectable early in embryonic development, spiking later before dropping [28,29]. HuD interacts with various mRNAs, including those involved in protein synthesis and growth factors. A previous study has demonstrated that HuD interacts with Satb1 to regulate Neurod1, a protein critical for retinal cell growth [30,31].

**Figure 1:**
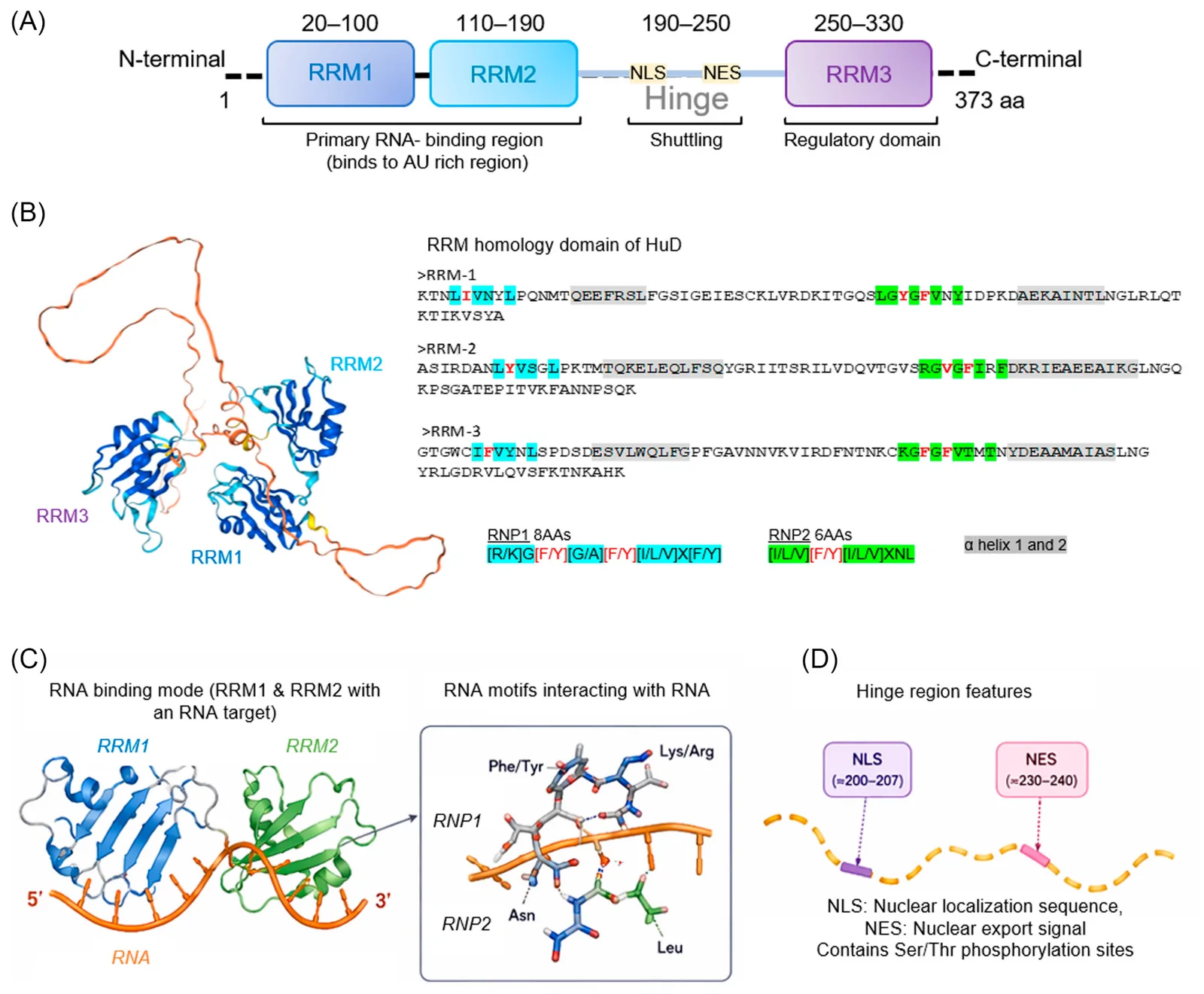
(**A**) The three RRM domains in the 373 amino acid generalized HuD protein structure are RRM1 and RRM2, which are close proximally, and RRM3, which is at the distal point and joined by a hinge region. RRM3 is the regulatory domain of the HuD protein, while RRM1 and 2 function as RNA-binding domains that bind to RNAs with AU-rich sequences. (**B**) The RRM domains in the HuD structure were retrieved from the AlphaFold Protein Structure Database (https://alphafold.ebi.ac.uk/; viewed on 4 June 2026). Sequence of RRM domains with RNP1 and 2 regions were marked in blue and green color. Red amino acids are important and mostly responsible for RNA attachments. (**C**) RNA binding mode: AU-rich RNA sequences are accommodated by a cleft formed by RRM1 and 2. RNP motifs interact with RNA via hydrogen bonds, electrostatic interactions, and Thr/Lys amino acids. (**D**) Because the hinge region has Ser/Thr phosphorylation sites that enable HuD to enter and exit the nucleus, it is essential for HuD nuclear localization and nuclear export.

The HuD protein comprises three highly conserved RNA recognition motifs (RRM1, RRM2, and RRM3) arranged so that RRM1 and RRM2 form a tandem unit at the N-terminus, followed by a flexible hinge region and a more functionally varied RRM3 at the C-terminus [32] (Figure 1A). RRMs follow a standard β1-α1-β2-β3-α2-β4 fold, forming a β-sheet surface that binds to RNA via conserved RNP1 and RNP2 motifs (Figure 1B). This allows for stacking and hydrogen-bonding interactions with AU-rich regions in target mRNAs. Functionally, RRM1 and RRM2 work together to produce high-affinity and sequence-specific RNA binding, resulting in a stable RNA–protein interface (Figure 1C), whereas RRM3 contributes to additional RNA interactions as well as protein–protein interactions that govern translation [33]. The intervening hinge region is fundamentally disordered and serves a vital regulatory role by limiting nuclear localization (Figure 1D). Structurally, partial crystallographic and NMR investigations show that the tandem RRMs create a compact RNA-binding platform, whereas RRM3 is more flexible, allowing interaction with other regulatory partners [34,35]. This structural arrangement allows HuD to stabilize target mRNAs, regulate their translation, and support neuron-specific processes, including differentiation and synaptic plasticity, setting it apart from comparable family members like ELAVL1.

HuD contributes to the maintenance of particular mRNAs in adult brains, promoting the development of stem cells into neurons. Knockout mice lacking HuD show an increase in neural progenitor cells but a decrease in early neuronal differentiation, indicating that HuD regulates many phases of neurodevelopment [5,36]. This protein’s binding promotes the stability of ACHE mRNA during neural development while also preventing CDKN1A mRNA from breaking down [37]. In later stages, HuD appears to inhibit neural progenitor cell growth while promoting neuronal differentiation [36].

According to research, HuD is required not only for neuron production but also for their function, survival, learning, memory, and adaptation following injury. HuD is located in growing axons in developing neurons as well. It promotes axon and dendrite growth in mature neurons. HuD promotes neurite development and the creation of dendritic connections, which are essential for brain networking. It helps neurons develop and survive by stabilizing GAP43 mRNA. It increases the translation of BDNF mRNA, particularly after nerve damage, which may aid in nerve repair [7,36,38,39]. Moreover, HuD affects the stability and expression of a large number of target mRNAs that are important for neuronal health. According to studies, HuD promotes regeneration in damaged axons, while HuD silencing reduces axon growth, in turn causing problems with dendritic growth and motor neuronal activity [7,8,32,40,41].

HuD is also present in neurons associated with learning and memory, such as those in the hippocampus [42,43,44]. It may also play a role in neurodegenerative diseases like Parkinson’s and Alzheimer’s, as well as motor neuron disorders including spinal muscular atrophy and amyotrophic lateral sclerosis. Genetic polymorphisms in HuD have been connected to the development of Parkinson’s. Other studies suggest that it helps to stabilize certain RNAs that may contribute to Alzheimer’s pathogenesis [28,45,46]. HuD modulates gene levels and synaptic connection formation in motor neurons [47,48], highlighting its involvement in many neurodevelopmental processes and possible therapeutic target.

## 4. Molecular Functions of HuD in Carcinology Context

**Figure 2:**
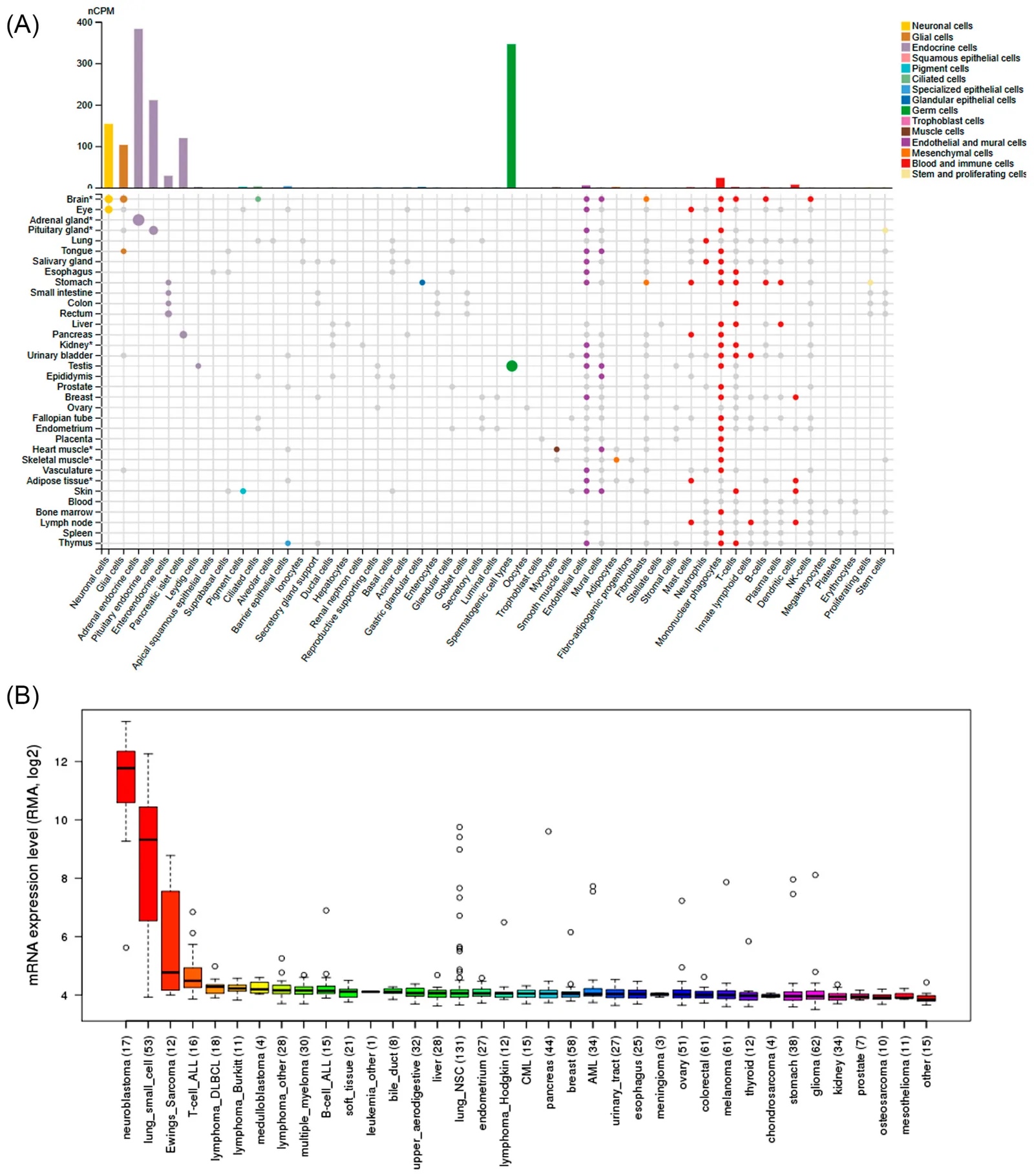

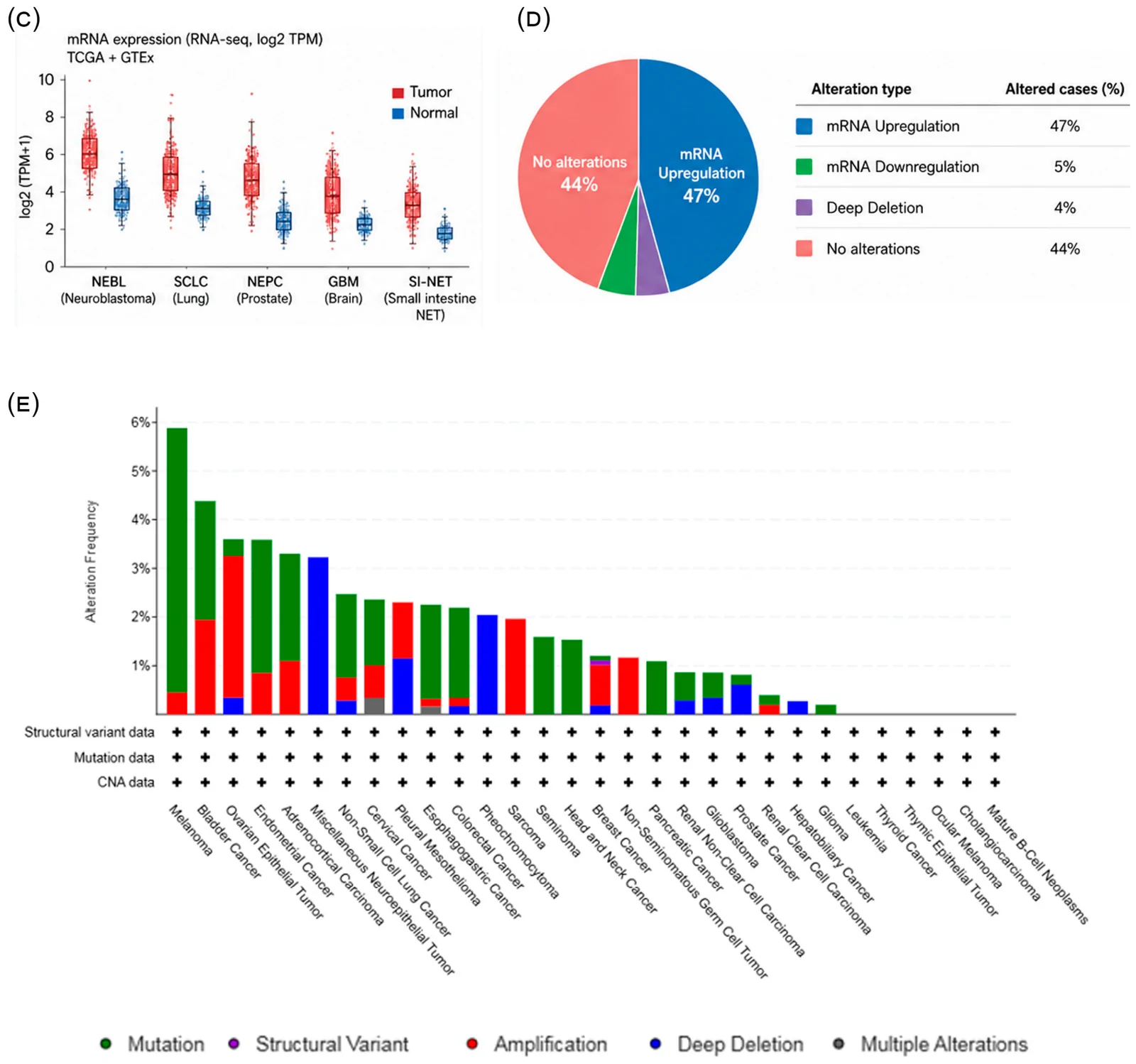
HuD expression in several tissues and malignancies. (**A**) HuD expression was found in cells with immunological and neurological origins. The information was obtained from https://www.proteinatlas.org/ (accessed on 15 July 2026). The expression patterns were represented in the histograms and the dots represent the cell types that express HuD in the specific tissue. A summary of normalized single cell RNA (nCPM) from all single cell types. Color-coding is based on cell type groups, each consisting of cell types with functional features in common. * Single nuclei. (**B**) A histogram was created by observing the HuD expression in different tumors. The two malignancies with the greatest expression levels were small cell lung cancer and neuroblastoma. The information was taken from https://sites.broadinstitute.org/ccle (accessed on 10 July 2026). (**C**) HuD expression comparison between normal and cancerous tissues. (**D**) Data pertaining to HuD mutations were acquired from https://www.cbioportal.org/ (evaluated on 10 July 2026). (**E**) Types of HuD genetic changes in cancer (TCGA pan-cancer). Instead of DNA-level mutations, HuD abnormalities in cancer are primarily caused by variations in expression. Data was taken from the TCGA pan-cancer atlas studies at https://www.cbioportal.org/ (accessed on 10 July 2026).

HuD is becoming more widely recognized as an important regulator of stress-adaptive survival programs in neuroendocrine tumors. While aberrantly expressed in cancers such as small cell lung cancer (SCLC) or other neuroendocrine malignancies (Figure 2), it rewires post-transcriptional gene regulation in ways that promote survival under adverse conditions such as hypoxia, oxidative stress, and nutritional constraints. A significant mechanism is HuD’s ability to stabilize mRNAs that encode stress-response and pro-survival proteins. These include transcripts associated with anti-apoptotic mechanisms (such as BCL2 family members), cell cycle regulation, and DNA damage repair [49,50]. HuD supports continuous protein production by extending the half-life of certain mRNAs, even when global transcription is inhibited during stressful conditions [19]. This forms a buffering system that permits tumor cells to withstand chemotherapy-induced stress and microenvironmental obstacles.

HuD also contributes to metabolic and signaling flexibility. It can speed up the translation of proteins involved in adaptive pathways like the PI3K/AKT and MAPK signaling cascades, which are essential for cell survival and proliferation [51,52]. In neuroendocrine tumors, where rapid growth and changing oxygen levels are common, post-transcriptional control facilitates dynamic switching between proliferative and quiescent phases, allowing cells to avoid apoptosis [53,54]; another significant element is its involvement in preserving the neuroendocrine phenotype [55,56]. HuD regulates neuron-specific genes that strengthen lineage identity, which is related to treatment resistance in malignancies such as SCLC [57]. HuD indirectly promotes tumor persistence by maintaining these lineage-defining mRNAs, limiting the efficacy of treatments that induce differentiation or cell death.

Under stress, HuD may bind to stress granules, such as cytoplasmic clumps of halted translation pre-initiation complexes, where it selectively maintains and later reactivates important mRNAs [7,17,58]. This activity strengthens tumor resilience by coordinating a quick recovery of protein production when favorable conditions resume. Overall, HuD functions as a post-transcriptional hub, combining stress signals with gene expression control to promote tumor cell survival, adaptability, and resistance in neuroendocrine tumors. As a result, it is being investigated as a possible therapeutic target: altering HuD-mRNA interactions or their downstream networks may sensitize tumors to stress and could enhance treatment outcomes.

In non-neural cells that undergo neoplastic neuroendocrine differentiation, the ELAVL4 gene becomes aberrantly reactivated, making it a valuable lineage-specific diagnostic marker. Mechanistically, HuD heavily correlates with tumor aggressiveness and poor clinical prognosis; for example, it is vastly overexpressed in small cell lung cancer (SCLC)—a high-grade pulmonary NEC—where it actively binds and stabilizes oncogenic transcripts like PFN2 and long noncoding RNAs to accelerate tumor progression. Furthermore, in stress-laden tumor microenvironments, upregulated HuD promotes tumor cell survival and therapeutic escape by stabilizing downstream transcripts such as GRB-10, which suppresses hyperactive mTORC1 signaling to induce protective autophagy and grant resistance against metabolic stressors [7,56]. Beyond its internal cellular roles, the profound overexpression of ELAVL4/HuD in neuroendocrine malignancies can spark severe autoimmune paraneoplastic neurological syndromes, as the host immune system generates anti-Hu antibodies that cross-react with the central and peripheral nervous systems [7,56].

HuD’s normal expression is limited to neurons and neuroendocrine cells. Because it is isolated within the immunologically privileged neurological system behind the blood–brain barrier, the systemic immune system does not come into contact with it normally. HuD, on the other hand, acts as a “tumor-testis antigen”-like molecule (or onconeural antigen) when it is abnormally or ectopically expressed by peripheral malignancies, most notably SCLC and neuroblastomas [59]. This aberrant systemic exposure disrupts immunological self-tolerance, which is typically caused by tumor-induced changes such as isoaspartylation, which make the protein appear foreign. This exposure triggers a powerful, dual-edged immune response: high-affinity anti-Hu cytotoxic T-cells and antibodies efficiently target and suppress the main tumor, but they also cross the blood–brain barrier and destroy healthy neurons.

## 5. HuD and Its Mutations for Onset of Neuroendocrine Tumors

Mutations in the HuD gene during early development can cause temporary nerve issues and reduced cell growth. Mice lacking HuD exhibit strong hindlimb reflexes but do not experience substantial neuron loss [47]. While DNA sequencing programs have revealed a variety of somatic mutations in HuD (including missense, nonsense, and splice site modifications) in malignancies such as lung, ovarian, and colorectal, they are not considered primary causes [60]. However, subgroups of neuroendocrine lung tumors have shown inactivating mutations and regulatory region alterations.

A prior investigation of lung neuroendocrine tumor samples revealed two types of somatic mutations: one was a stop codon mutation that resulted in a truncated protein, and the other produced a frameshift [57]. Several questionable nucleotide alterations were also observed, including loss of heterozygosity (LOH) at the HuD locus, which was present in multiple SCLC tumors, indicating that both gene copies were inactive [57,61]. The mutations affect either the RRM 2 domain or the region between RRM 2 and 3 (hinge region). Previous studies suggest that the first two RRM domains aid in the binding of short-lived mRNAs, but the hinge and third RRM may be important in the binding of longer mRNA tails [7,32]. These identified mutations impair the protein’s normal functioning. The identification of inactivating mutations in HuD in the majority of neuroendocrine tumors is surprising, given that HuD is normally expressed in these cancers [57,62]. HuD expression levels varied across SCLC cell lines with differing neuroendocrine development, implying that these mutations may reflect various molecular subgroups of tumors rather than being associated with autoimmune reactions.

There are numerous biologically feasible answers for this seeming paradox. HuD may have context-dependent actions, with effects varying between tumor initiation and tumor maintenance, SCLC molecular subtypes, and untreated versus treatment-exposed cancers. Alternatively, HuD’s deletion could be a passenger event linked to specific genetic backgrounds or lineage states. It is also plausible that HuD supports survival only in a subset of SCLC cells, but its absence gives an advantage in another cellular environment. However, each of these options would necessitate explicit research and cannot be simply assumed in support of an oncogenic hypothesis.

Similar vigilance is required when interpreting prognostic information. Prognostic association and oncogenic causality are not synonymous. If high HuD expression is associated with poor survival, this may indicate a link between HuD and aggressive disease, but it does not explain the prevalence of recurring loss-of-function changes. Conversely, if HuD deletion is associated with a poor prognosis, it could imply that HuD loss leads to aggressive disease, directly challenging a basic paradigm in which HuD activity drives cancer. In-depth research with HuD in this context is still necessary for an improved understanding of the subject.

## 6. HuD Induces Proliferation, Migration, and Invasion in SCLC

HuD is important in nerve damage and neuropathies, especially in the genesis of neuroendocrine tumors. It is present in all samples of neuroblastoma and SCLC [63,64]. There has been investigation into the genetic mechanisms that drive ectopic nELAV gene expression and their influence on neuroendocrine lung cancers, particularly SCLCs. HuD, LYPLAL1-DT, miR-204-5p, and PFN2 are all involved in the growth, migration, and spread of SCLC. The role of the lncRNA LYPLAL1-DT, which is abundant in SCLC, was validated using functions in both live and laboratory trials [56]. The interactions of HuD/LYPLAL1-DT and HuD/PFN2 have also been verified. The association of LYPLAL1-DT with miR-204-5p, which ultimately leads to increased PFN2 levels during SCLC growth, was investigated [56].

The study also looked into why LYPLAL1-DT levels are high in SCLC and demonstrated strong HuD expression in SCLC, which supports previous findings. HuD stabilizes LYPLAL1-DT, boosting cell proliferation, motility, and dissemination, demonstrating its oncogenic involvement, according to a novel method. HuD regulates PFN2, which is necessary for cancer growth and actin reorganization. HuD was discovered to raise PFN2 levels by direct engagement. Certain limitations were acknowledged, such as the small number of SCLC samples utilized to establish the regulatory pathways and the difficulty in assessing LYPLAL1-DT linkages due to a lack of data. The optimal therapy target within the HuD/LYPLAL1-DT/miR-204-5p/PFN2 pathway remains unknown.

## 7. High Expression of HuD in Cancer Cells Endures in Several Stress Environments

HuD helps cancer cells survive stress, particularly in aggressive malignancies such as neuroblastoma and small cell lung cancer (SCLC). HuD helps cells survive and form tumors by stabilizing specific mRNA targets. Cancer cells experience increased metabolic and oxidative stress in stressful settings. HuD combats this through many major mechanisms:

1. Autophagy Activation: In neuroblastoma, HuD stabilizes mRNAs that inhibit mTORC1 activation, resulting in autophagy. This process enables cells to recycle components and receive nutrients required for survival [19].
2. Apoptosis Suppression: HuD stabilizes proteins that prevent planned cell death, allowing cancer cells to survive even when under great stress [7,19].
3. Tumor Proliferation: In SCLC, HuD stabilizes RNAs that promote tumor development and spread [56].

Clinically, high HuD levels are generally associated with a poor prognosis and more aggressive tumors, but HuD could also be a therapeutic target. Reducing HuD activity in cancer models greatly reduces tumor survival and growth. In some situations, the immune system may mistakenly attack damaged HuD proteins, causing neurological disorders.

## 8. HuD Promotes Tumor Cell Survival in Neuroblastoma Through Antiapoptotic and Autophagy Signaling

**Figure 3:**
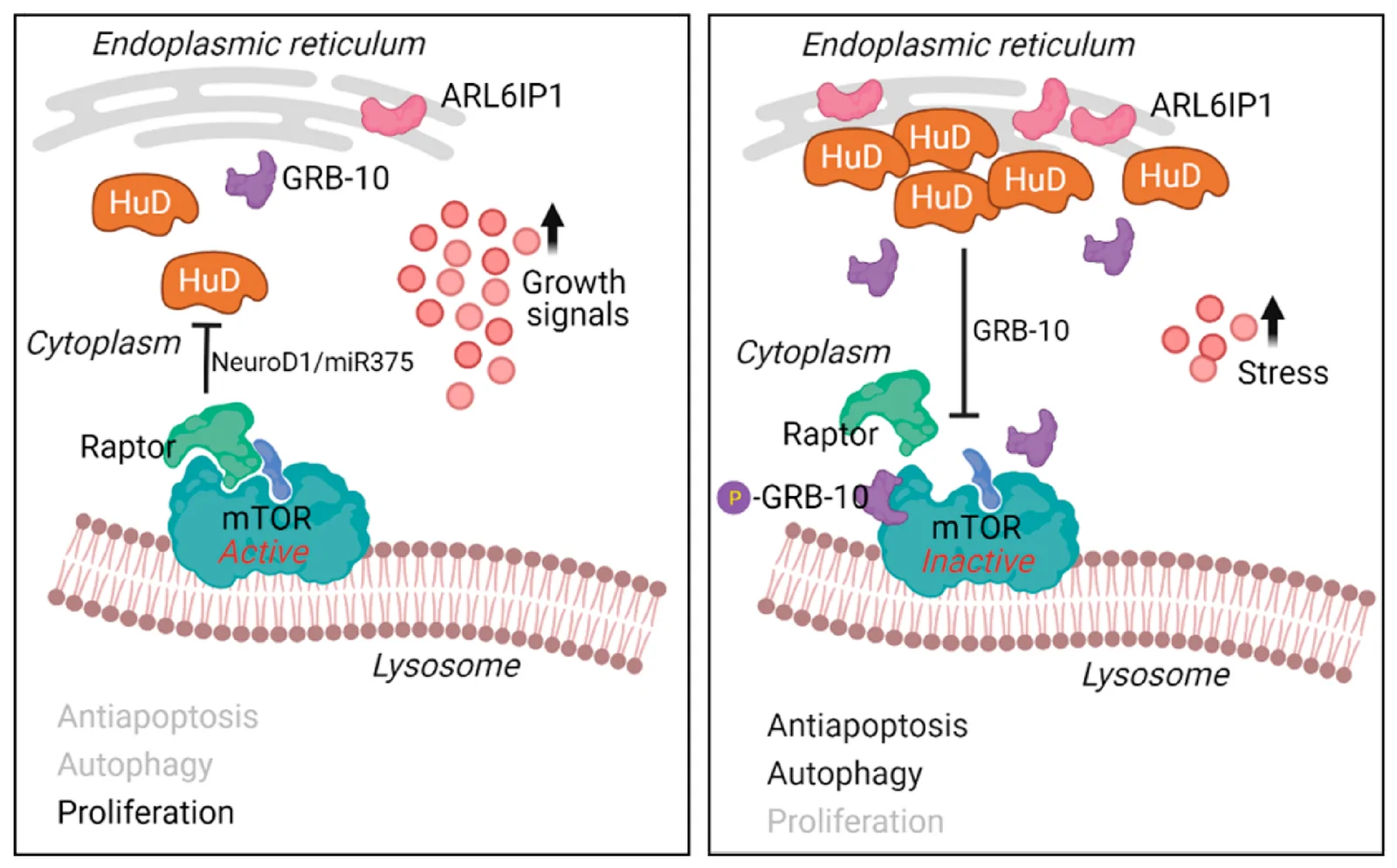
Pro-survival signals in neuroblastoma are provided by overexpressed HuD. Through the NeuroD1/miR375 signaling, mTORC1 suppresses HuD expression under optimal growth circumstances or in the presence of potent growth cues. Elevated active mTORC1 induces the transcription factor NeuroD1 level, which in turn generates anti-HuD miR375. Alternatively, under various stress conditions, the mTORC1 block on HuD lifts. GRB-10 and ARL6IP1 RNA signals are bound to and stabilized by elevated HuD levels. Raptor binding to mTORC1 is still inhibited by elevated GRB-10 levels, which lowers mTORC1 activity and releases autophagy repression. More pro-survival messages are sent by elevated ARL6IP1 levels. A transmembrane ER-shaping protein called ARL6IP1 stops apoptosis by obstructing the caspase pathway. (BioRender.com was used to make the diagram).

HuD is identified as a novel autophagy activator that inhibits the activity of the mTORC1 kinase. HuD inhibits mTORC1 through GRB-10 and improves cell survival against apoptosis via ARL6IP1 (Figure 3). In stressful environments, HuD promotes autophagy and provides antiapoptotic signals, notably in cancer cells. Experiments showed that silencing HuD lowered the mRNA levels of its substrates, GRB-10 and ARL6IP1, which can bind to and stabilize HuD [19].

HuD is necessary for tumor growth in neuroblastoma; lowering HuD resulted in decreased tumor growth. The mTORC1 pathway regulates protein translation and signaling via downstream substrates such as 4E-BP1 and S6K1, as well as inhibiting autophagy by phosphorylating ULK1. When GRB-10 is phosphorylated, it separates Raptor, the positive mTOR regulator, from the mTORC1 complex. Changing GRB-10 levels altered mTORC1 activity, as evidenced by changes in phosphorylated S6 levels and Raptor associations [65], indicating that HuD modulates mTORC1 inhibition on autophagy. Because mTORC1 can inhibit autophagy by phosphorylating ULK1 and the transcription factor TFEB [66], overexpression of HuD resulted in increased autophagy markers. Furthermore, a regulatory circuit exists in which HuD receives signals from NeuroD1 and miR375 via mTORC1 activity, implying a balance between HuD and mTORC1 signals based on external inputs such as nutrition and internal ATP availability.

**Figure 4:**
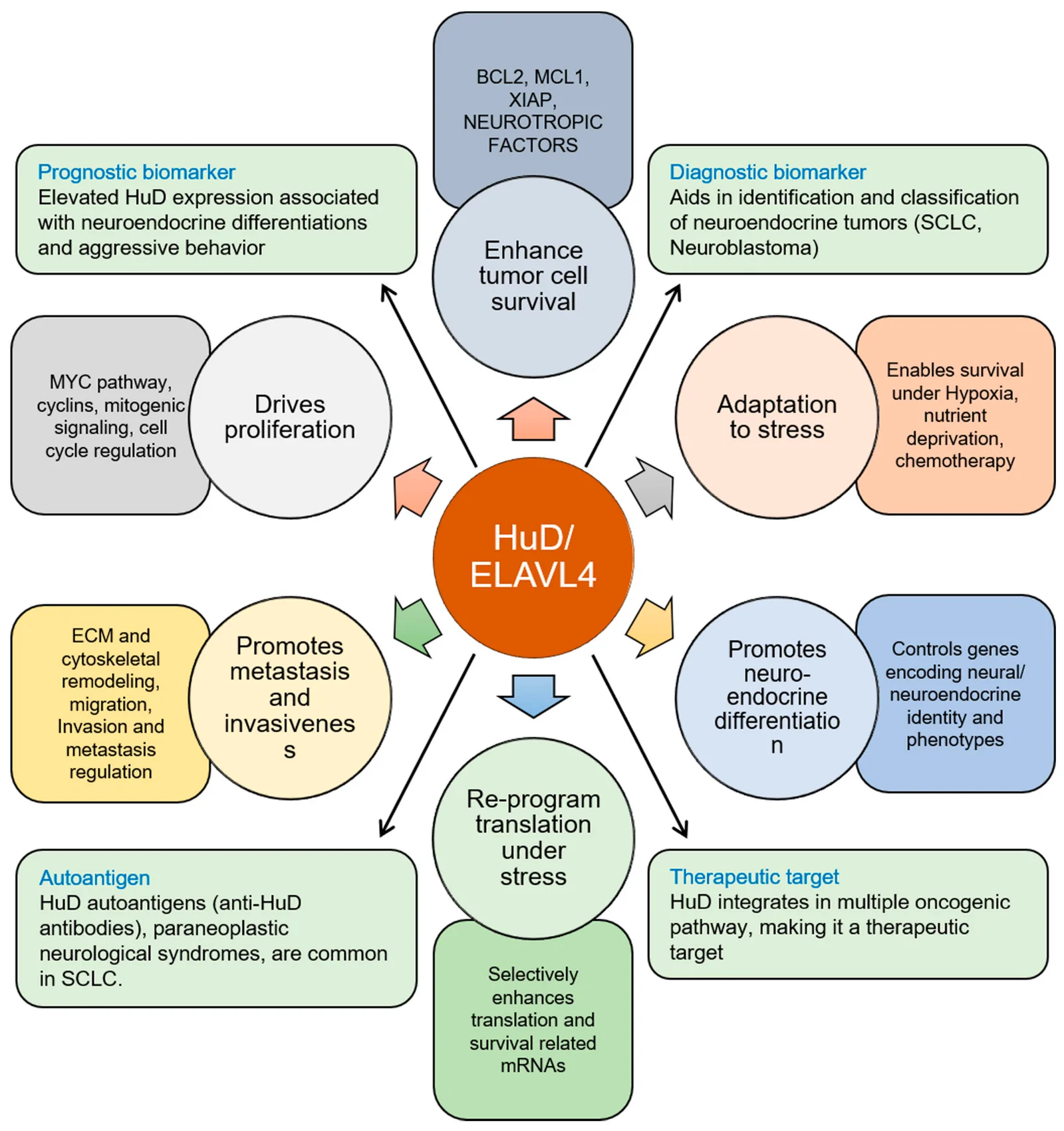
HuD’s function in the various stages of cancer development was outlined. We found that HuD was in charge of preserving oncogenicity and helping malignant cells adjust to the demanding conditions in the tumor microenvironment.

There have been reports of anti-HuD autoantibodies in certain neuroblastoma and SCLC patients, which are often associated with smaller tumors and longer survival rates [61,64]. The immunological response to isomerized surface HuD could play a role in these cancer instances. HuD’s negative control of mTOR contrasts with prior findings that HuD supports mTOR in neurons. HuD, although lowering mTOR activity, also stabilizes critical mTOR RNA targets, implying that it plays a role in cancer cell survival under stress via several mechanisms (Figure 4).

HuD acts on XIAP (X-linked inhibitor of apoptosis protein) and other targets to enhance tumor development, treatment resistance, and cellular survival [7,67]. HuD has been demonstrated to interact with the mTORC1 pathway, regulating critical apoptotic regulators in the endoplasmic reticulum (e.g., ARL6IP1) [19]. Both Hu proteins aid tumor cells in avoiding programmed cell death (apoptosis) by sponging miRNAs and maintaining critical pro-survival mRNAs [68,69]. XIAP is the primary regulator of cell death. Its principal function in cancer is to block both intrinsic and extrinsic apoptotic pathways by physically attaching to and inhibiting caspases 3, 7, and 9. Many types of cancer, including neuroblastoma, acute myeloid leukemia, and solid tumors, have high levels of XIAP.

Nerve fibers can infiltrate the tumor stroma, which promotes aggressive tumor growth and spread [70,71].

HuD is generally limited to the neurological system; therefore, its ectopic expression in neuroendocrine tumors (such as small cell lung cancer) causes an immune response. The resultant autoantibodies attack brain cells, resulting in severe autoimmune neurological consequences known as paraneoplastic syndrome [63,72]. Alternatively, a certain increase in local BDNF production activates tropomyosin receptor kinases (Trk), notably TrkB, resulting in powerful downstream oncogenic signals via the PI3K/Akt and Ras/Raf/MEK/ERK pathways [73]. This cascade significantly enhances tumor growth, epithelial-to-mesenchymal transition (EMT), and resistance to treatment. Inhibiting HuD-mRNA connections or blocking downstream Trk receptors reduces tumor growth and increases cancer cell susceptibility to stress.

HuD commonly hijacks neurotrophic factor and receptor transcripts, stabilizing and upregulating them. This relationship enhances cancer cell survival, proliferation, and tumor-nerve communication, allowing aggressive malignancies to avoid stress and thrive in the tumor microenvironment (TME). HuD binds directly to BDNF, NGF, and NT-3 [38,39,74]. HuD improves their local translation by reducing transcript degradation. HuD acts as an oncogenic driver in malignancies such as neuroblastoma when exposed to adverse conditions (e.g., nutritional depletion or hypoxia). It stabilizes particular stress-response transcripts, activating autophagy and pro-survival pathways (such as mTOR) in the absence of conventional nutritional sensors, protecting tumor cells from apoptosis. HuD promotes the recruitment of the kinase Akt to translation sites, resulting in a functional adaptor complex that stimulates cellular proliferation and tumor migration [51,52,75]. The HuD-mediated overexpression of neurotrophic factors promotes bidirectional nerve-tumor interactions. Tumors use neurotrophin signaling to induce nerve fiber infiltration into the tumor stroma, which promotes aggressive tumor growth and spread [70,71].

## 9. HuD Promotes Metastasis and Invasion in Cancer

HuD promotes cancer growth, dissemination, and invasion in numerous forms. It stabilizes cancer-related mRNAs, aids in the development of cell structures, and regulates matrix metalloproteinases (Figure 4). HuD promotes PFN2 mRNA stability in small cell lung cancer (SCLC), resulting in enhanced cell mobility and invasion [56].

Apart from NETs, HuD showed an association with oral squamous cell carcinoma (OSCC), where it was associated with cancer severity and spread because they increase the levels of growth factors and enzymes that promote invasion. HuD expression was assessed in 82 cases of OSCC. It occurred in 36.6% of instances and was associated with characteristics such as tissue type, lymph node spread, and invasion mechanism [76]. HuD levels were much higher in aggressive HSC3 cells than in less aggressive HSC4 cells. HuD was found to alter the expression of VEGF-A, VEGF-D, MMP-2, and MMP-9, highlighting its potential as a diagnostic and therapy target in OSCC [76].

## 10. HuD Reprograms Translation Under Stress

Under stress, neurons produce more of the RNA-binding protein HuD to help them survive. HuD binds to the 3′ UTR of particular mRNAs, stabilizing them, preventing early degradation, and enhancing translation. This mechanism is critical for dealing with problems such as hypoxia, cellular damage, and low-nutrient conditions (Figure 4).

HuD also aids in the assembly of stress granules under high levels of stress. It travels to the cytoplasm, where it interacts with other proteins to produce ribonucleoproteins [77,78,79]. This activity inhibits overall protein production while protecting essential survival-related mRNAs from destruction. Furthermore, HuD improves the translation of brain-specific proteins by directly delivering translation factors to mRNAs, particularly when regular protein synthesis pathways are ineffective. It also aids in metabolic reprogramming during nutritional stress by boosting the translation of critical factors required for energy metabolism and cell health. HuD concentrates on mRNAs that are important for fast recovery after stress, such as those that promote nerve growth and defend against cell damage. However, in disorders like ALS, Alzheimer’s, and Parkinson’s, this protective response fails, resulting in toxic protein clumps that damage neurons.

## 11. Context-Dependent Functions of HuD in Different Tumor Types

**Figure 5:**
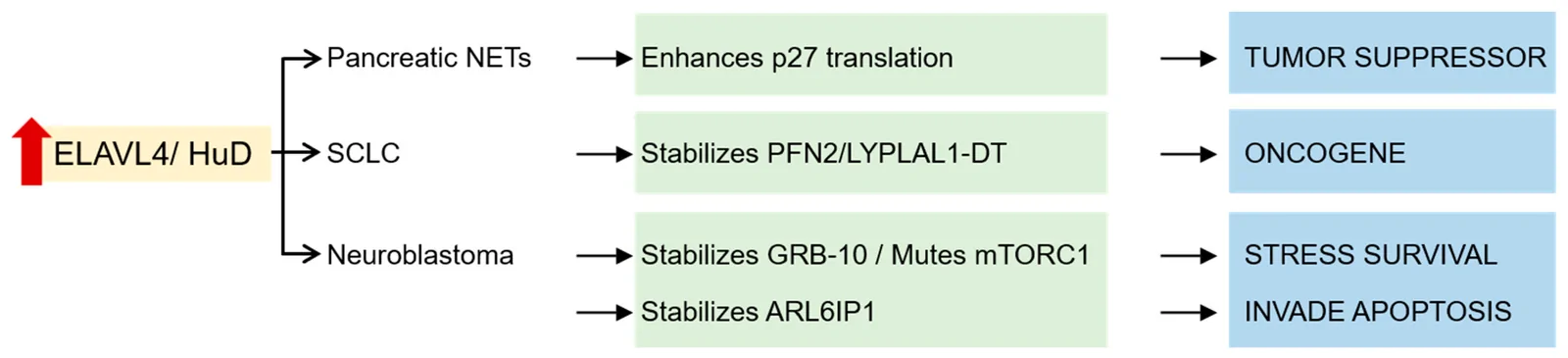
HuD/ELAVL4 appears best viewed as a context-dependent RNA regulator whose oncogenic potential emerges when its neuronal RNA-regulatory program is co-opted by malignant cells.

We observed that although HuD has a higher expression pattern in multiple cancer types, in several cancer types HuD expression was deliberately lower, which is correlated with better progression of those cancers. Therefore, HuD appears best viewed as a context-dependent RNA regulator whose oncogenic potential emerges when its neuronal RNA-regulatory program is co-opted by malignant cells. Its role may be particularly important in neuroendocrine cancers, where neuronal differentiation programs, cellular stress, and therapeutic resistance intersect. Determining the HuD–RNA interactome in individual tumor types, together with functional perturbation and single-cell analyses, will be critical for establishing whether HuD is a driver, passenger, biomarker, or therapeutic vulnerability in each cancer context (Figure 5).

### 11.1. Tumor Suppressor in Pancreatic Neuroendocrine Tumors (pNETs)

In well-differentiated pancreatic NETs, HuD acts explicitly as a tumor suppressor by binding concurrently to p27 mRNA, which enhances its translation and induces cell-cycle arrest. Clinical data show that a loss of HuD expression in pNET patients correlates with decreased p27 levels, increased tumor size, higher WHO grade, angioinvasion, and shorter progression-free survival.

### 11.2. Pro-Tumorigenic Oncogene in Small Cell Lung Cancer (SCLC)

Conversely, in high-grade pulmonary carcinomas like SCLC, HuD is heavily overexpressed and acts as a powerful driver of tumorigenesis. In this setting, it binds and stabilizes the actin-binding transcript PFN2 and the long non-coding RNA LYPLAL1-DT, directly promoting cytoskeleton remodeling, rapid cell proliferation, migration, and invasive capacity.

### 11.3. Stress Adaptation Factor in Neuroblastoma

In pediatric neuroblastoma, HuD functions as a metabolic survival rheostat. Under microenvironmental stress (such as chemotherapy or nutrient deprivation), HuD stabilizes GRB-10 and ARL6IP1 mRNAs. Upregulated GRB-10 interacts with and dampens the hyperactive mTORC1 complex, shifting the cancer cell away from apoptosis and into protective, survival-sustaining autophagy.

## 12. Molecular Mechanisms Regulating HuD

HuD is associated with neurological disorders and plays an important role in neuronal development and plasticity. It raises questions regarding the processes that regulate this protein and how it is expressed. Currently, only a few researchers have looked at the mechanisms that regulate HuD at various levels. More extensive research into these molecular processes is required.

### 12.1. Post-Translational Control of HuD Expression and Function

In certain instances, such as those caused by growth hormones and cellular stress, HuD expression can be affected, but the underlying mechanisms are mostly unclear. Protein kinase C (PKC), coactivator-associated arginine methyltransferase 1 (CARM1), and protein kinase B (PKB/AKT) are the three most well-known pathways that impact HuD.

Research indicates that PKCα can boost nELAV levels and facilitate their movement to the nucleus in particular cells [80]. It also increases the interaction and phosphorylation of nELAV proteins, which influences their position inside neurons and aids in the stabilization of mRNA associated with neuronal development. When PKCε and HuD are active, they lead HuD to be phosphorylated at various locations. HuD then plays an important role in nerve cell development pathways [80,81,82].

CARM1, a methyltransferase, has also been found to impact HuD. It can methylate certain amino acids on HuD, affecting its mRNA stability [83]. When CARM1 activity is lowered, mRNA levels associated with cell differentiation increase, indicating that CARM1 activity is critical for controlling the shift from cell proliferation to differentiation. CARM1’s reduced activity has an effect on other mRNAs, implying that pathways other than HuD methylation may regulate them. Furthermore, a recent study found that PKC and CARM1 have opposite impacts on HuD and cell formation. In some neurons, activating PKC decreases CARM1’s capacity to methylate HuD, allowing HuD to bind to target mRNAs and promote neuron development [45,74]. This shows that PKC activation can have a negative effect on CARM1 activity, hence advancing neuron growth.

The phosphatidylinositol 3-kinase (PI3K)/AKT1 pathway also promotes HuD function during the translation stage. AKT1 physically interacts with HuD. Furthermore, prolonged activation of AKT1 raises HuD levels, showing that it increases HuD abundance and promotes neuronal development [48]. Overall, activating distinct signaling pathways can impact HuD’s involvement in keeping cells growing or urging them to differentiate into neurons.

### 12.2. Post-Transcriptional Control of HuD Expression

There is evidence that trans-acting factors influence HuD mRNA metabolism. For example, miR-375 has been demonstrated to target HuD’s 3′ UTR and reduce its levels by influencing mRNA stability and translation, hence reducing neurite outgrowth [84]. In neuroblastoma, activation of the transcription factor NeuroD1 via mTROC1 elevates miR-375 levels [19]. That, in turn, regulates HuD activity in cancer cells in the presence of excessive nutrients. Overall, multiple regulatory proteins and microRNAs appear to fine-tune HuD transcripts.

### 12.3. Transcriptional Control of HuD Expression

The only factors known to regulate HuD transcription were thyroid hormone (T3) and FoxO1, both of which have a negative effect on HuD levels. T3 was found to reduce HuD mRNA in N2A mouse neuroblastoma cells, and its levels were negatively related to HuD mRNA in the rat brain [85]. In low glucose conditions, FoxO1 inhibits HuD transcription. This suggests that these variables may maintain low HuD levels in nonneuronal and stem cells [48,86]. HuD is activated during early neuronal development in P19 cells via neuron-specific transcriptional regulation. Neurogenin 2 (Ngn2) targets a conserved 400 bp region in HuD E-boxes. Ngn2 binds to these E-boxes, which activates HuD expression during neuronal development [87]. Ngn2 is essential for neurogenesis and plays roles in learning, memory, and neuronal disorders [88], suggesting its crucial role in HuD transcription during neuronal development.

### 12.4. Strategies for Therapeutic Targeting in Tumors That Overexpress HuD

**Figure 6:**
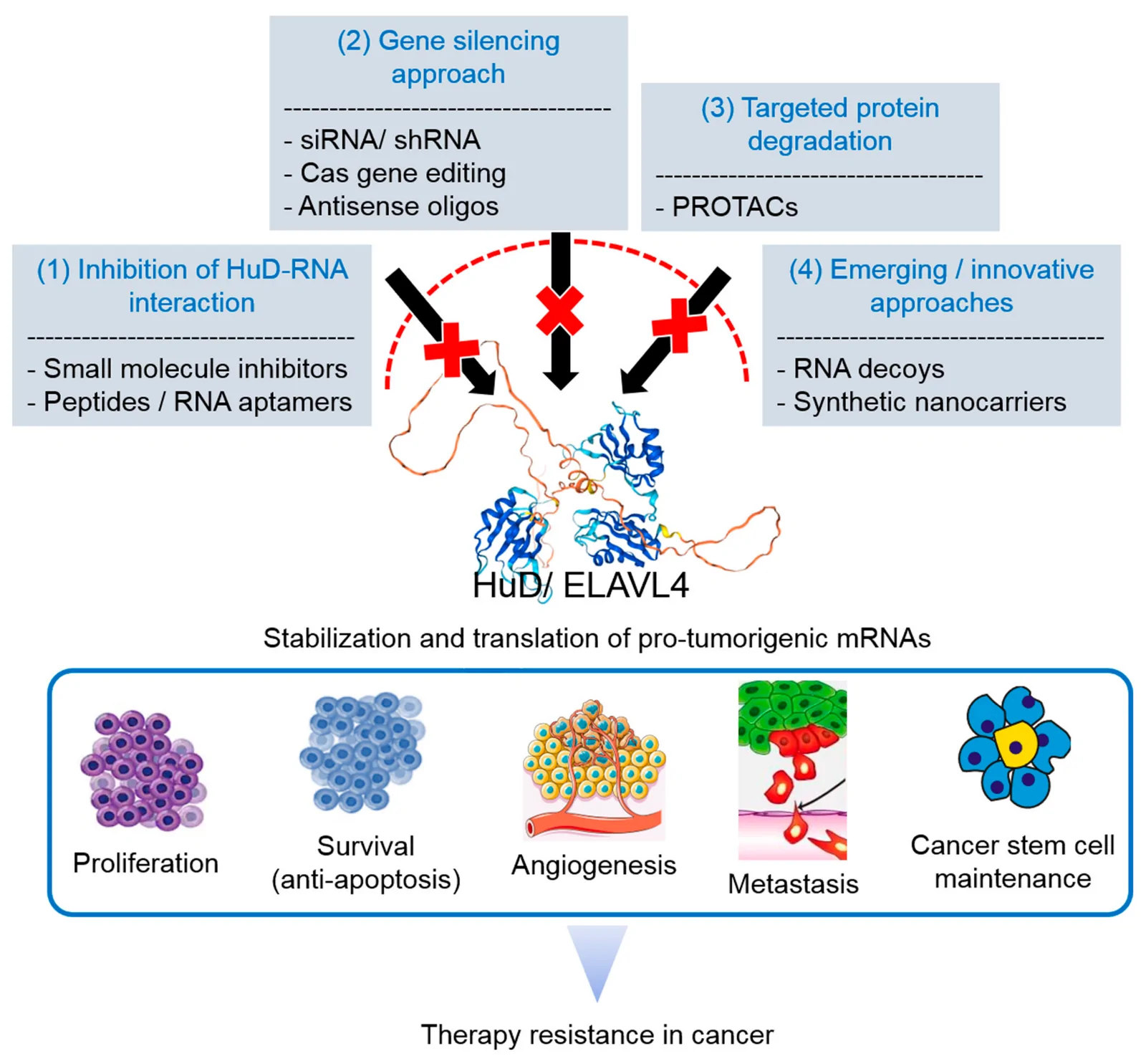
Targeting HuD may prove to be an effective strategy for treating a variety of malignancies that show high levels of HuD. We outlined four different methods that can be applied to this cancer cell for targeting HuD to block its activity in the cell; arrow with red color cross represents the application of different methods to inhibit HuD’s activity.

Due to its crucial function in controlling post-transcriptional gene expression, which supports tumor growth, survival, metastasis, and therapeutic resistance, HuD overexpression in a number of cancers, especially neuroendocrine tumors and small-cell lung cancer, has become a promising therapeutic target (Figure 6). HuD is an RNA-binding protein whose RNA-binding activity is a promising therapeutic target because it stabilizes and improves the translation of many mRNAs involved in cell cycle progression, anti-apoptotic signaling, stress adaptation, angiogenesis, epithelial–mesenchymal transition, and cancer stem cell maintenance [19,89,90]. One of the most promising methods is the structure-guided synthesis of small-molecule inhibitors that can interfere with the interaction between HuD and target transcripts that include AU-rich elements; nevertheless, achieving high selectivity for RNA-binding proteins is still a significant challenge.

Gene-silencing technologies, such as small interfering RNA (siRNA), short hairpin RNA (shRNA), antisense oligonucleotides (ASOs), and CRISPR/Cas-mediated genome editing, provide alternative options for suppressing HuD expression and have demonstrated promising preclinical results. Proteolysis-targeting chimeras (PROTACs) have emerged as a novel platform in recent times [91] to induce selective HuD degradation, possibly circumventing the limitations of conventional inhibitors.

For instance, predicting the structural confirmation of HuD by an artificial intelligence model, AlphaFold, may accurately anticipate the conformations of RBP complexes with diverse RNA substrates or small-molecule drugs, thereby advancing the elucidation of their binding specificity and dynamic regulation mechanisms. AI-powered virtual screening and binding free-energy calculations can help uncover small-molecule leads that disrupt HuD-RNA interactions or create PROTAC compounds that specifically degrade HuD. This organically blends structural biology, computational biology, and targeted therapy techniques, revealing new computationally assisted R&D avenues for precision interventions in neuroendocrine tumors [92]. Indeed, artificial intelligence has produced significant advances in medical research, diagnosis, and treatment.

Additionally, highly targeted therapy options may be provided by RNA aptamers and synthetic peptides designed to interfere with HuD-RNA or HuD-protein interactions. Given HuD’s physiological expression in the nervous system, effective and tumor-specific delivery of these agents via lipid nanoparticles, polymeric nanoparticles, or antibody-conjugated delivery systems will be essential to maximizing therapeutic efficacy while reducing off-target toxicity [93].

Combining HuD-targeted therapies with traditional chemotherapy, radiotherapy, targeted agents, or immune checkpoint inhibitors may result in synergistic antitumor effects and lessen therapeutic resistance because HuD aids cells in adapting to oxidative stress, hypoxia, endoplasmic reticulum stress, and chemotherapy-induced stress. Precision oncology may also be advanced by combining multi-omics analysis with biomarker-guided patient classification to determine who will benefit most from HuD-targeted treatments [94]. The transition of HuD-directed therapies from preclinical models to clinical trials is anticipated to be accelerated by ongoing advancements in RNA-targeted drug discovery, structural biology, medicinal chemistry, and targeted drug delivery, even though no HuD-specific therapies have yet entered routine clinical practice.

The proposed therapeutic strategy of systemic HuD suppression or degradation necessitates a more thorough examination of its therapeutic window. This is not just a delivery issue. HuD/ELAVL4 has an important physiological role in neurons, and the study explores its participation in neurological dysfunction and neurodegenerative illness. As a result, systemic siRNA, ASO, or PROTAC-mediated depletion highlights a key on-target toxicity concern: can adequate HuD suppression be obtained in tumor cells while retaining neuronal HuD activity essential for normal tissue homeostasis? The therapeutic section should thus consider tissue selectivity, CNS exposure, the magnitude and duration of target suppression, reversibility, and potential neurological damage rather than presuming that tumor-directed administration alone solves the problem.

A second, independent constraint arises from the genetic observations discussed above. Tumors with biallelic HuD inactivation, LOH, truncating, or frameshift mutations may have limited or no functional HuD accessible for therapeutic degradation. As a result, a HuD degrader would be inherently useless in tumors that have already lost the target, making functional HuD status a possible precondition for therapeutic response. This is especially critical if such mutations occur frequently in SCLC. Therefore, it is important to identify the expected responder population and explore if HuD genotype, HuD protein abundance, or functional HuD dependence should be used as indicators for patient selection. Taken together, these considerations substantially constrain the therapeutic scope of systemic HuD degradation and should be explicitly incorporated into the discussion rather than presenting HuD depletion as a broadly applicable therapeutic strategy.

### 12.5. Future Directions in HuD Research in Cancer

Future studies on HuD should concentrate on comprehending its context-dependent actions in various cancer types and identifying the molecular mechanisms behind its dual oncogenic and tumor-suppressive effects. A deeper comprehension of its post-transcriptional regulatory networks will be possible through thorough characterization of the HuD-regulated transcriptome using cutting-edge RNA–protein interaction technologies, such as enhanced crosslinking and immunoprecipitation sequencing (eCLIP), RNA immunoprecipitation sequencing (RIP-seq), and single-cell multi-omics. Understanding the mechanisms governing HuD’s activity and subcellular localization in cancer cells will be aided by more investigation into the regulation of HuD by non-coding RNAs, epi-transcriptomic modifications, and post-translational modifications like phosphorylation, ubiquitination, SUMOylation, and methylation.

Future studies should examine HuD’s function in oxidative stress, endoplasmic reticulum stress, hypoxia, nutritional restriction, and therapy-induced stress, as these pathways may contribute to tumor survival and therapeutic resistance, given the mounting evidence linking HuD to cellular stress adaptation. In order to evaluate HuD’s potential as a predictor of immunotherapy response, it is also crucial to determine how it influences the tumor’s immunological behavior, including immune checkpoint regulation, cytokine signaling, antigen presentation, and immune-cell infiltration. A promising path in precision oncology is the development of selective HuD-targeted medicines, such as small-molecule RNA-binding inhibitors, antisense oligonucleotides, siRNA-based techniques, PROTAC-mediated protein degradation, RNA aptamers, and nanoparticle-based delivery systems.

## 13. Concluding Remarks

HuD-driven molecular subtypes and therapeutically actionable vulnerabilities can be found concurrently by merging artificial intelligence and systems biology methodologies with genomes, transcriptomics, proteomics, epi-transcriptomics, metabolomics, and spatial transcriptomics. Translating mechanistic discoveries into clinical applications will depend on validating HuD as a diagnostic, prognostic, and predictive biomarker through extensive multicenter clinical studies and creating reliable preclinical models like genetically modified mice, patient-derived organoids, and xenografts. In the end, these interdisciplinary initiatives are probably going to establish HuD as a crucial regulator of post-transcriptional gene expression in cancer, as well as a potential therapeutic and diagnostic target for customized cancer treatment.

## Data Availability

No new data were created or analyzed in this study. Data sharing is not applicable to this article.
